# *Bacopa monnieri* as an Antioxidant Therapy to Reduce Oxidative Stress in the Aging Brain

**DOI:** 10.1155/2015/615384

**Published:** 2015-08-27

**Authors:** Tamara Simpson, Matthew Pase, Con Stough

**Affiliations:** ^1^Centre for Human Psychopharmacology, Swinburne University of Technology, Hawthorn, VIC 3122, Australia; ^2^Department of Neurology, Boston University School of Medicine and Framingham Heart Study, Boston, MA 02118, USA

## Abstract

The detrimental effect of neuronal cell death due to oxidative stress and mitochondrial dysfunction has been implicated in age-related cognitive decline and neurodegenerative disorders such as Alzheimer's disease. The Indian herb *Bacopa monnieri* is a dietary antioxidant, with animal and *in vitro* studies indicating several modes of action that may protect the brain against oxidative damage. In parallel, several studies using the CDRI08 extract have shown that extracts of *Bacopa monnieri* improve cognitive function in humans. The biological mechanisms of this cognitive enhancement are unknown. In this review we discuss the animal studies and *in vivo* evidence for *Bacopa monnieri* as a potential therapeutic antioxidant to reduce oxidative stress and improve cognitive function. We suggest that future studies incorporate neuroimaging particularly magnetic resonance spectroscopy into their randomized controlled trials to better understand whether changes in antioxidant status *in vivo* cause improvements in cognitive function.

## 1. Introduction

The world's population is aging rapidly [1]. One consequence of an aging population is an increased prevalence of chronic, age-related illnesses and disorders involving oxidative stress and low level chronic inflammation [2]. Increasing age is a major risk factor for dementia, including Alzheimer's disease (AD), and other prevalent neurodegenerative disorders [3]. The causes of brain aging and dementia are complex and incompletely understood.

Oxidative stress is one mechanism that detrimentally contributes to the aging process and is inextricably linked to neurodegenerative disorders [4]. Interventions that manipulate the oxidative stress mechanisms may decrease oxidative damage, slow the rate of aging, and lessen the risk of neurodegenerative disorders, increasing the lifespan of older adults. Research has begun to focus on developing effective health and lifestyle interventions so that older adults are able to remain both physically and cognitively healthy into older age, reducing the social and economic burden associated with an aging population [5].

The Indian herb,* Bacopa monnieri* (EBm) may serve as a dietary antioxidant, with several modes of action to protect the brain against oxidative damage and age-related cognitive decline. Several studies using the standardized CDRI08 extract have shown that EBm improves cognitive function particularly in the elderly [6–8]. Animal and* in vitro *studies using the standardized extract CDRI08 have revealed promising results to elucidate EBm's antioxidant properties (e.g., [9–12]). The aim of this review is to examine the evidence for EBm as a potential therapeutic antioxidant to reduce oxidative stress in the aging brain and as a mechanism by which it may improve cognition. We also discuss magnetic resonance spectroscopy (MRS) as a technique to elucidate the antioxidant mechanisms of action of EBm in human research* in vivo.*

## 2. The Aging Brain

Deterioration in memory performance is a signature of advancing age. Almost 50% of adults aged 64 years and over tend to report difficulties with their memory [13]. In addition to memory, executive function, processing speed, attention, and spatial ability have also been shown to deteriorate with age [14–19]. While most cognitive functions decline with age, cognitive aging does not occur uniformly at the same rate and to the same extent for all people [20]. There may be several reasons for this including differences in lifestyle factors, particularly dietary intake.

Aging is a predominant risk factor for dementia, including AD [3]. An imbalance between the production and clearance of abnormal proteins called *β*-amyloid [21], the formation of neurofibrillary tangles, and neuroinflammation are hallmarks of advanced brain aging and dementia [22]. Neuroimaging studies have reliably identified that with increasing age, ventricular enlargement, white matter hyperintensities, reduction in gross brain volume, reductions in frontal and temporoparietal volume, and higher levels of cortical atrophy occur in the brain [23]. The shrinkage of cortical volume is believed to impact cognitive functioning negatively, reducing a person's cognitive ability [24]. Functional magnetic resonance imaging studies have revealed that age-related memory changes may be due to altered activation of the prefrontal cortex (PFC). For example, compared to their younger counterparts, older adults recruit a broader area of the PFC due to bilateral activation of this region [25]. Cabeza [26] conceptualized this phenomenon as a reduction in hemispheric asymmetry, a compensatory response to a loss in neural efficiency. Other researchers explain older adult's broad brain activation during working memory tasks with the compensation-related utilization of neural circuits hypothesis (CRUNCH) [27]. This hypothesis suggests that the compensatory response of the brain's bilateral over activation occurs due to older adults recruiting maximal neuronal resources earlier than younger adults when completing the same tasks, thereby leaving no neuronal resources left for a higher load of difficulty, resulting in poorer performance. Since these two theories, researchers believe the compensatory response is in fact a protective scaffolding effect to support or prop up underlying adverse factors associated with brain ageing like brain shrinkage, reduction in dopamine receptors, neural inefficiency, noise, or both (scaffolding theory of aging and cognition (STAC)) [28]. To enable researchers to predict cognitive status and change over time, a revised STAC model (STAC-r) incorporates aging and life experience factors like exercise and cognitive training that influence structure and function of the aging brain which in turn may enhance or deplete neural resources [29].

More recently, neuroimaging studies have investigated the correlation between brain neurometabolite levels, as an indication of underlying molecular or cellular changes that may be related to aging. The technique of MRS is a noninvasive method of obtaining biochemical information about body tissue [30]. MRS has been used to study age-related degenerative diseases like cognitive impairment and AD [31, 32] and neuropsychiatric disorders like depression [33] and schizophrenia [34]. MRS can be utilized for early detection of disease and for monitoring medical therapies or treatments [35]. Changes in metabolites are purported to reflect changes in different brain indices such as neuronal viability/function (N-acetyl-aspartate; NAA), cellular turnover (Choline; Cho), metabolic activity (glutamate, glutamine; GLX), inflammation in the brain (myo-inositol; Myo), and oxidative stress (glutathione; GSH) [36].

MRS studies have investigated the correlation between changing brain neurometabolite levels and cognitive performance in healthy aging populations [37, 38]. A study by Ross and colleagues [39] identified significant correlations between the integrity of frontal white matter NAA metabolite and cognitive function represented by processing speed, visual memory, and attention tasks with a healthy elderly cohort. A large study conducted by Ferguson and colleagues [40] investigated the relationships between NAA, Cho, and Cr and cognitive function in a group of healthy elderly men. Positive correlations were found between NAA/Cr and Cho/Cr ratios with test measures of logical and verbal memory. The authors postulated that high levels of Cr are the best predictor of poor cognitive performance. An increase in the Cr signal has been reliably identified in healthy elderly brains compared with their younger counterparts [41–43]. These studies support the premise that MRS is a valid technique to measure subclinical changes in cognition across the lifespan.

Alternatively, researchers investigating MRS metabolite markers in clinical cohorts with Alzheimer's disease (AD) have reliably found NAA to be lower and Myo to be higher when compared to cognitively healthy older adults (e.g. [31, 41]). However, inconsistent Cho levels have been identified with some studies reporting an increase in Cho of people with AD [44] while others have not [45]. Collectively, these physiological, pathophysiological, and structural changes that occur with increasing age highlight the complex nature of the aging brain.

Understanding the mechanisms and role of oxidative stress in the aging process is currently considered to be important to elucidate the key to longevity. An emerging theory in the literature postulates that the balance between oxidation/reduction reactions (redox state) within cells is important for healthy aging [46]. If there is a disruption to the mechanisms of redox state (impaired signalling and regulation), then age-associated functional losses will occur [46]. Oxidative stress, antioxidants, and the aging brain will be discussed below.

## 3. Oxidative Stress, Antioxidants, and the Aging Brain

### 3.1. Oxidative Stress Mechanisms

**Figure 1:**
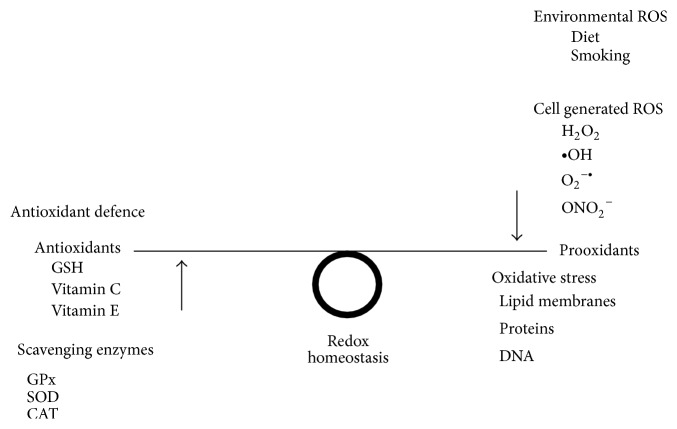
*Oxidative stress mechanisms*. GSH (glutathione) and vitamin C (ascorbic acid) and vitamin E (*α*-tocopherol) are nonenzymatic antioxidants that assist in antioxidant defence against reactive oxygen species (ROS), to inhibit or repair damage to cells. Scavenging enzymes GPx (glutathione peroxidase), SOD (superoxide dismutase), and CAT (catalase) work to prevent oxidative damage by detoxifying reactive oxygen species (ROS). Environmental ROS as well as cell generated ROS like H_2_O_2 _(hydrogen peroxide), ∙OH (hydroxyl free radical), O_2_^−∙^ (superoxide anion), and ONO_2_^−^ (peroxynitrite) are all prooxidants that when in abundance can lead to an imbalance in the redox homeostasis causing oxidative stress, which have detrimental effects to lipid membranes, proteins, and DNA.

Although oxygen is needed for survival, the brain is sensitive to oxygen metabolic activity that produces ROS [47]. Approximately 95%–98% of ROS such as hydrogen peroxide (H_2_O_2_), hydroxyl free radical (∙OH), superoxide anion (O_2_^−∙^), and peroxynitrite (ONO_2_^−^) are formed in mitochondria as by-products of cellular respiration [48]. Studies of mitochondria isolated from the brain indicate that 2–5% of the total oxygen consumed produces ROS [49]. An imbalance between prooxidant and antioxidant reactions occurs when the equilibrium between the beneficial and harmful effects (redox homeostasis) is interrupted ([50]; refer to Figure 1). This imbalance produces a steady accumulation of oxidative damage in macromolecules that increase with aging, causing a progressive loss in cellular function and efficiency of processes [51]. Free radicals in the brain are responsible for significant harmful effects to cellular function and damage to DNA, proteins, membrane lipids, and components of mitochondria [47, 52]. The brain is particularly sensitive to free radical damage due to its high metabolic rate, concentration of unsaturated fatty acids, cytotoxic actions of glutamate and reduced antioxidant systems with a lower activity of glutathione peroxidase (GPx) and catalase (CAT) compared to other organs [9, 53].

Aging decreases the ability of the brain to scavenge free radicals, thus decreasing available antioxidants, particularly the most abundant endogenous antioxidant GSH [47, 50]. There is a delicate balance between the positive and negative effects of free radicals. In a normal physiological state, fluctuation in ROS production is balanced with ROS scavenging capacity [54]. Oxidative stress occurs when ROS production exceeds that of ROS scavenging capacity. Oxidative stress is a significant feature of aging, most likely due to a combination of reduced ROS scavenging capacity, impaired redox state, and increased ROS production [55]. This imbalance in the cellular redox mechanisms may contribute to the slow onset and progressive nature of neurodegenerative diseases, as well as age-related cognitive decline [46, 56]. Severe, extensive, or more prolonged oxidative damage is highly toxic and these toxic effects contribute significantly to the aging process [57, 58].

### 3.2. Antioxidant Mechanisms

The human body has an innate defence mechanism consisting of endogenous antioxidants to negate the detrimental effects of oxidants [59]. Antioxidants have the ability to reduce oxidative stress in the body by scavenging ROS to either inhibit or repair damage. Antioxidant enzymes superoxide dismutase (SOD), CAT, GPx, and glutathione reductase (GR) present the first line of defence against free radical damage under conditions of oxidative stress [60, 61]. Nonenzymatic antioxidants, glutathione (GSH), vitamin C (ascorbic acid), and vitamin E (*α*-tocopherol) are all phenolic compounds that offer protection by altering oxidants to either nonradical end products or transporting radicals to areas where their effects will be less damaging [56]. Vitamins A, C, and E, selenium, and coenzyme Q10 effect important antioxidant actions to protect neural tissue from “attack” by free radicals [62]. Eating a varied diet can provide a mixture of oxidants and antioxidants. Fruits and vegetables rich in vitamins A, C, and E provide a healthy defence against the formation of free radicals. Such fruits and vegetables also increase the number of cell receptors available for antioxidant enzyme action [47]. Dietary polyphenols with antioxidant properties have protective effects against many degenerative diseases including diabetes, cancer, and cardiovascular diseases aiding in the prevention of oxidative stress [63, 64].

**Figure 2:**
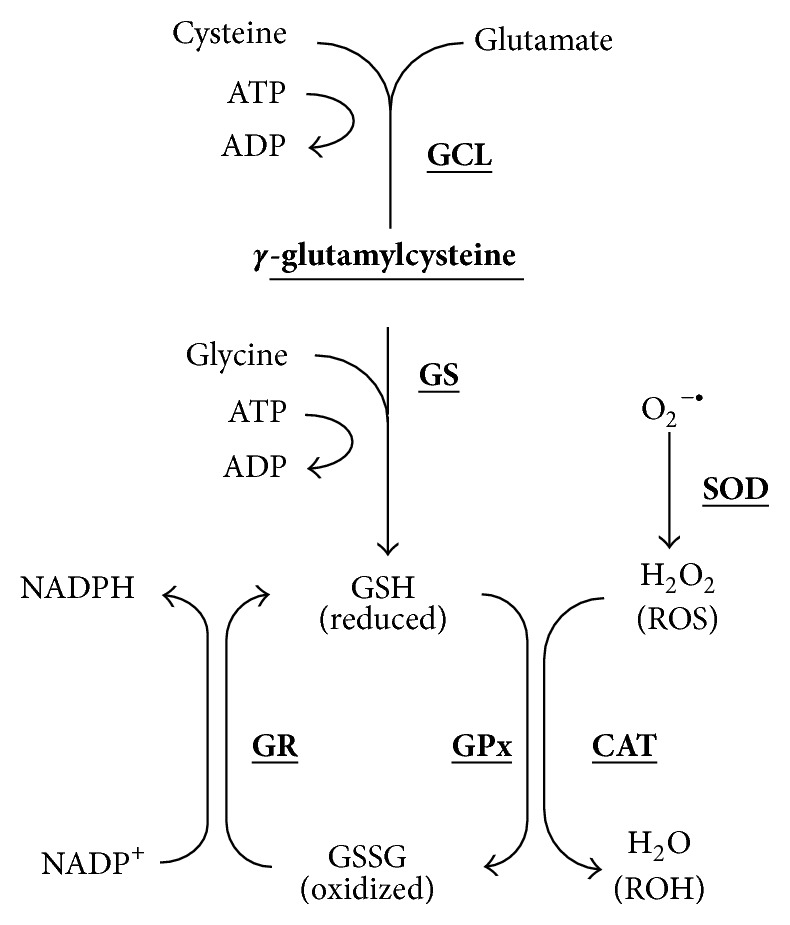
*Glutathione redox cycle*. Glutathione (GSH) is synthesized from the amino acids glutamate (Glu), cysteine (Cys), and glycine (Gly) in a two-step pathway requiring energy from ATP. Glu and Cys are combined via the action of glutamate cysteine ligase (GCL). This dipeptide then combines with Gly via a reaction from glutathione synthetase (GS). GSH undergoes a redox reaction using glutathione peroxidase GPx to detoxify reactive oxygen species (ROS) like hydrogen peroxide (H_2_O_2_). The main source of H_2_O_2_ is from the conversion of superoxide anion (O_2_^−∙^) by the enzymatic action of superoxide dismutase (SOD). GSH is converted to an oxidized form (GSSG) and is recycled back to GSH by the enzymatic reaction of glutathione reductase (GR) which requires the cofactor nicotinamide adenine dinucleotide phosphate (NADPH) to form a redox cycle.* NB:* bold and underlined text represents enzymes.

The GSH redox cycle lowers H_2_O_2_ levels, thus lowering the formation of damaging hydroxyl radicals ([65]; refer to Figure 2). GSH is a tripeptide (L-*γ*-glutamyl-L-cysteinylglycine) found everywhere within the cells of the body. It is involved in many physiological functions. GSH is responsible for detoxifying ROS into nontoxic substances (water and oxygen) [66] and is critical for the maintenance of normal function and neuronal survival [67]. It is involved in the synthesis of proteins and DNA, enzyme activity, metabolism, transport, and cell protection [66]. GSH is oxidised to glutathione disulphide (GSSG) resulting in intracellular redox imbalance which is reflected in a decreased GSH to GSSG ratio, often referred to as oxidative stress [68]. GSH levels in tissue decrease with age [69]. Impaired GSH metabolism has been implicated in the pathogenesis of clinical mental disorders like schizophrenia and bipolar disorder [70] as well as neurodegenerative disorders including AD [31] and Parkinson's disease [71]. It is not surprising then that oxidative stress generated by ROS is consistently linked to these conditions.

A study conducted by Berger and colleagues [72] used MRS to investigate* in vivo* GSH levels before and after the administration of an omega-3 fatty acid (ethyl eicosapentaenoic acid; E-EPA) within patients who had experienced their first episode of psychosis. Supplementation with E-EPA was reported to increase GSH concentration by 38% in the temporal lobe of these patients. More importantly, the increase in GSH correlated with improvement in negative symptoms. This promising result provides support for further research to be conducted to elucidate the effect of other supplements on cerebral GSH levels in normal and clinical populations.

GSH levels, quantified using MRS, have been used to investigate oxidative stress in different brain regions (frontal cortex, parietal cortex, hippocampus, and cerebellum) of healthy adults and in the bilateral frontal cortices of patients with mild cognitive impairment and AD [32]. In healthy females compared to healthy males, mean GSH levels were higher (left frontal cortex, *P* = 0.006; right posterior cortex, *P* = 0.01) and that GSH distribution was different between the hemispheres for females and males. GSH levels were significantly depleted in the right frontal cortex of female AD patients compared to healthy female participants (*P* = 0.003), whereas, for males, the left frontal cortex was significantly depleted (*P* = 0.05) when comparing healthy males to AD patients. GSH was lower in mild cognitive impaired patients compared to healthy participants, but the difference was not statistically significant. GSH is therefore an important biomarker of redox state which can be monitored to investigate disease progression or normal age-related changes [32].

Despite the compelling research linking brain metabolite alterations to changes in cognitive function with age and changes in GSH levels in clinical populations, to date, no studies have incorporated the use of MRS as a technique to measure metabolite changes in response to EBm. Clinical trials have not investigated the antioxidant defence system, particularly targeting the ubiquitous antioxidant, GSH, in response to EBm using MRS. Finally, clinical trials have not examined the cognitive correlates of MRS after the chronic administration of EBm.

Altering the inefficiency of the antioxidant system by boosting the redox potential with thiols, particularly the ubiquitous antioxidant, GSH, may be a way to reduce age-associated decline in functional abilities. As discussed below, the administration of EBm may improve the antioxidant redox state, thus leading to improved functional outcomes such as enhanced cognitive performance.

## 4. What Is* Bacopa monnieri*?

*Bacopa monnieri* (Linn), commonly referred to as “Brahmi,” from the plant family Scrophulariaceae is a creeping herb found in India and neighbouring tropical countries that grows in wet marshland up to 1500 m in altitude [73]. It has been traditionally used in Ayurvedic medicine to treat conditions such as fever, inflammation, pain, asthma, epilepsy, and memory decline [10]. It has been used in a standardized form in clinical research since 1996 [74]. Steroidal saponins and Bacosides A and B are the active chemical constituents responsible for improving both learning and memory [75, 76]. Other constituents include bacopasaponins D, E and F as well as alkaloids, flavonoids, and phytosterols [77, 78]. Some of the chemical constituents of EBm are lipophilic [79, 80]. This means that they can combine with or dissolve in lipids giving them the ability to cross the blood-brain barrier. Bacosides are believed to repair damaged neurons by enhancing kinase activity and neuronal synthesis linked with the restoration of synaptic activity, culminating in the improvement of nerve impulse transmission [81]. Antidepressant and anxiolytic effects have been reported in animal studies [82, 83] although conflicting findings have been reported in human trials [6, 7, 84, 85]. However it is the memory enhancing effects of EBm that have generated the most attention [86]. Various mechanisms may be involved in the neuroprotective and memory enhancing effects of EBm, such as the binding and detoxification of metal ions [87], free radical scavenging [88], or increasing antioxidant activity [9]. Animal models have shown that EBm can exert vasorelaxant [89], adaptogenic [90], anti-inflammatory [91], metal ion chelating [92], and cholinergic modulatory effects [93]. Neuroprotective effects have been identified in animal models of epilepsy [94] and amnesia [95] as well as reducing ischemia-induced memory deficits in rats [96]. EBm also appears to inhibit numerous *β*-amyloid oxidative stress pathways involved in AD pathology [92] and antioxidant properties related to GSH redox state [97]. The role of oxidative stress and alterations in the antioxidant GSH redox state in response to EBm will be expanded upon below.

## 5. Antioxidant/Oxidative Stress Mechanisms of Bacopa

The antioxidant properties of EBm are widely recognised and have been discussed in various reviews [10, 98, 99]. Several histological (*in vitro*) and animal studies have established that EBm bacosides or extract improve the system's defences against oxidative stress by decreasing the formation of free radical accumulation in the brain. In an early study investigating the antioxidant activity of EBm, lipid peroxidation in the prefrontal cortex, striatum, and hippocampus of rats was inhibited. Bhattacharya and colleagues [9] found a dose related increase in enzyme activity responsible for scavenging reactive oxygen species, namely, SOD, CAT, and GPx in these brain regions of rats after 14 and 21 days of chronic administration of EBm. Interestingly, the same study compared the antioxidant effects of the drug deprenyl, which also improved antioxidant enzyme activity, but only in the prefrontal cortex and striatum of the rats and not the hippocampus. They suggested that this increase in the free radical scavenging activity of bacosides may be responsible for facilitating the cognitive action of EBm. Similarly, in a different study, the modulation of antioxidant activity in diabetic rats was again through a significant increase in SOD, CAT, GPx, and GSH levels showing a significant reversal of redox imbalance and peroxidative damage to enhance the defence system against ROS [11]. Other studies also support a free radical scavenging mechanism in response to EBm [75] by reducing the formation of free radicals [92, 100]. In addition, a more recent study found that a EBm ethanolic extract was able to adjust the level of endogenous oxidative markers in various brain regions of prepubertal mice [101].

Furthermore, an* in vitro *study by Russo and colleagues [88] investigated H_2_O_2_ induced cytotoxicity and DNA damage in human nonimmortalized fibroblast cells in response to an ethanol extract of EBm. They also investigated the free radical scavenging capacity and the effect on DNA cleavage induced by H_2_O_2_. EBm was able to inhibit superoxide anion formation in a dose dependent manner, indicative of free radical scavenging ability and a protective effect was observed against H_2_O_2_ cytotoxicity and DNA damage. A more recent* in vivo* and* in vitro* study conducted by Shinomol and colleagues [102] used 3-nitropropionic acid (NPA), a fungal toxin that causes neurotoxicity in both animals and humans, in comparison with the effects of an ethanolic extract of EBm in the mitochondria of the striatum of rats and dopaminergic (N27) cells. As predicted, the NPA caused oxidative stress in the mitochondria of the striatum, while pretreatment with EBm prevented NPA oxidative reactions and reduction of reduced GSH and thiol levels.

In experimental models of ischemia, diabetes and aluminium and cigarette induced toxicity, pretreatment with EBm (40 mg/kg/day to 250 mg/kg/day) and Bacoside A (10 mg/kg/day) has been identified to prevent lipid peroxidation and play a role in antioxidant activities by modulating the effects of enzymes (Hsp 70, cytochrome P450, and SOD in the rat brain) known to be involved in the production and scavenging of ROS, resulting in antistress activity in rats [103–105]. Again, these studies support the premise that EBm has the ability to restore antioxidant defence mechanisms and protect against the adverse effects of peroxidative damage. EBm has also been shown to either exert antioxidant effects through metal chelation at the initiation level of the free radical chain reaction by chelating ferrous ions, or be attributed to the detoxification of free radicals at the propagation level [87, 92]. In another study in rats, the effect of EBm detoxified ROS ONO_2_^−^ in astrocytes [106].

Cumulatively, animal and* in vitro *studies provide support for antioxidant mechanisms of EBm. Animal and* in vitro* studies have identified that GSH is particularly useful to examine antioxidant capacity and changes in oxidative stress. Taking into account the findings of the studies described above, EBm may increase the cellular inefficiency of the antioxidant system by boosting the redox potential with GSH (e.g., [9, 11]). In turn, the administration of EBm as a therapeutic intervention may be a way to reduce age-associated decline in functional abilities. The therapeutic properties of plants like EBm have generated much scientific investigation due to their compelling antioxidant properties, little to no side effects, and economic sustainability [107].

## 6. Clinical and Research Implications

Oxidative stress plays a role in aging and neurodegenerative disorders. Based on the animal* in vitro* and* in vivo* studies discussed in this review, EBm can be utilized as a therapeutic strategy against oxidative damage and cognitive decline in the elderly. Supplementation with EBm is likely to support the antioxidant defence pathways altering the redox status, which are vital components for normal functioning, while improving cognitive ability. Given that with age it is believed that the antioxidant system is compromised and GSH levels are reduced, EBm has the potential as a therapeutic antioxidant to reduce oxidative stress and improve cognitive performance.

## 7. Future Directions

While the central role of oxidative stress in age-related cognitive decline and neurodegenerative diseases has driven studies to examine the potential antioxidant benefits of EBm, studies have not incorporated* in vivo* brain imaging techniques to systematically study brain aging and central oxidative stress. MRS may be a useful technique to understand the antioxidant mechanisms, particularly studying GSH ROS detoxification,* in vivo*, as a result of EBm supplementation. Applying neuroimaging research techniques is important to be able to understand the* in vivo* effects underpinning the cognitive changes due to EBm. Future studies should consider the application of brain imaging modalities, particularly MRS, to be able to extend results beyond the explanation of mood, general health, and cognitive behavioural outcomes in response to dietary supplementation in human randomized clinical trials.

## 8. Conclusion

Further exploration into the complex mechanisms of action of EBm in nutritional aging studies may reveal promising insights into antioxidant metabolic changes, supporting dietary nutritional supplementation for therapeutic means. This review has described how EBm has the potential as a therapeutic antioxidant to reduce oxidative stress, a mechanism that may be responsible for improving cognitive performance and offer neuroprotection. Employing the neuroimaging technique of MRS to investigate GSH antioxidant levels may be useful to elucidate the mechanisms of action underlying the cognitive enhancing effects of EBm. Such research may also assist in our understanding of how to improve cognition in the elderly.
